# Time-sensitive healthcare guidelines for youth with chronic diseases in custody: gaps in care

**DOI:** 10.1038/s41390-023-02947-x

**Published:** 2023-12-09

**Authors:** Colin Dickens, Ahalya Ramesh, Temiloluwa Adanlawo, Michael R. DeBaun

**Affiliations:** Department of Pediatrics, Vanderbilt-Meharry Sickle Cell Disease Center of Excellence, Vanderbilt University Medical Center, Nashville, TN USA

## Abstract

**Case study:**

On May 9th, 2023, a U.S. Border Patrol detained a family of five near Brownsville, TX. During processing, one of the family members, an eight-year-old girl, ADRA, was noted to have sickle cell anemia and a heart disease condition. Five days after they arrived at the Donna Facility, on May 14th, ADRA displayed symptoms, including abdominal pain and fever, and tested positive for Influenza A. She was administered medication and transferred to a designated isolation unit at the Harlingen Border Patrol Station. Despite her deteriorating condition and her mother’s urgent requests for medical intervention, there were no documented consultations with an on-call physician or considerations for her transfer to a local hospital. On May 17th, ADRA’s health critically declined, marked by multiple visits to the medical unit for vomiting and abdominal pain. An ambulance was dispatched only after ADRA experienced a seizure and became unresponsive, Fig. 1. Her subsequent death was deemed a “preventable tragedy” attributed to systemic failures in the Border Patrol’s medical care and decision-making processes in a juvenile care monitor’s report.^1^

**Impact:**

This article adds to the existing literature by:

- Summarizing the gap in age-specific guidelines for six chronic diseases that occur in children and adolescents held in custody.
- Identifying the lack of adequate intervention strategies for acute management of chronic diseases for youth held in custody and strategies for improving health equity.

## Introduction

The American Pediatric Society’s President Issue of the Year is: *Improving quality health care for incarcerated youth and adolescents*.^2^ As part of this year-long initiative, our goal in this commentary is to describe the absence of medical guidelines for children and adolescents with chronic diseases held in jails, prisons, juvenile facilities, and immigration detention facilities (referred to subsequently as custodial settings). Evidenced-based guidelines acknowledging that our children’s chronic diseases exist in custodial settings is the first step in ensuring health equity for this near invisible, vulnerable, and prominent population of children and adolescents in the United States.

**Fig. 1:**
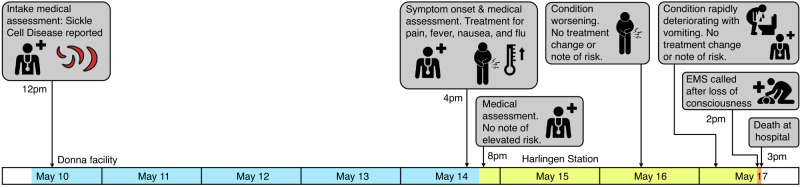
Timeline of death of a child with sickle cell disease and heart disease held in a detention facility. This timeline visualizes the events from an 8-year-old child’s detention by a U.S. Border Patrol, through her health deterioration, and to her subsequent preventable death, highlighting notable gaps in medical care and intervention responsiveness within the detention facilities.^1^

The health care for youth and adolescents held in custody remains challenging for health care providers. Annually, about 128,000 young individuals are admitted on delinquency charges.^3^ Compounding this issue, the number of unaccompanied minors at the U.S. southern border has reached unprecedented numbers, with over 131,519 unaccompanied children in the October 2022 to September 2023 fiscal year alone.^4^ The number of detained youths, coupled with the lack of clear and robust guidelines for clinical care management for children held in custody, presents a less-than-optimal healthcare environment.

The National Commission on Correctional Health Care (NCCHC) has established minimum health care standards for children and children in custodial settings.^5^ The NCCHC standards provide administrative and procedural guidance for delivering health care, for example, specifying the role and responsibilities of clinicians versus custodial staff and minimum required procedures for documentation, among many other recommendations. The NCCHC accreditation is not mandatory or published, and many states choose not to accredit services under NCCHC standards. The NCCHC’s mandate does not include developing evidence-based clinical guidelines for children and adolescents in custody but instead emphasizes the need for “clinical protocols [that] are consistent with national clinical practice guidelines”.^5^ Hence, the national clinical practice guidelines from the corresponding subspeciality-associated professional organizations are the standard for health care for youth held in custody. However, the age-specific subspecialty evidence guidelines for children held in custody are not commonly available.

The absence of age-specific guidelines for common life-threatening chronic diseases is a gap in improving child and adolescent health care. To address this gap, we identified six chronic diseases in children and adolescents associated with frequent exacerbations requiring prompt intervention to prevent lifelong sequelae: asthma, cystic fibrosis, type 1 diabetes, epilepsy, cancer, and sickle cell disease. We selected chronic diseases based on the observation that if a child or adolescent arrived in the Emergency Department with an acute exacerbation of their established chronic disease, the patient would have an emergency severity index of at least 2, a triage scale in a pediatric emergency department, indicating nursing triage within 20 minutes.^6^

## Existing clinical practice guidelines and role of sub-specialists

Among the selected six chronic diseases, only three specific evidence-based clinical guidelines addressed acute medical management of adults held in custody.^7–9^ No professional organization had pediatric-specific evidence-based clinical guidelines tailored to the needs of youth in custody, Table 1.^7–12^ This lack of age-specific guidelines for chronic disease care for youth highlights the need for further attention to the unique circumstances of the health needs of children and adolescents in custodial settings.

**Table 1:** Life-threatening chronic diseases with acute exacerbations.

Life Threatening Chronic Disease (acute exacerbation)	Time Sensitivity	Professional Organization	Guidelines For Adults in Custody Settings	Guidelines for Children and Adolescents in Custody
Type 1 Diabetes (hypoglycemia)	Immediately	American Diabetic Association	Yes	No
Asthma (status asthmaticus)	Immediately	Asthma and Allergy Foundation of America/American Thoracic Society	Yes/No	No
Cystic Fibrosis (acute pulmonary exacerbation)	Immediately	Cystic Fibrosis Foundation/American Thoracic Society	No	No
Sickle Cell Disease (acute vaso-occlusive pain)	Immediately	American Society of Hematology	No	No
Epilepsy (seizure)	Immediately	American Epilepsy Society	Yes	No
Cancer (acute illness)	Immediately	American Cancer Society	No	No

We have identified six chronic diseases where acute exacerbations of the underlying disease can result in a medical emergency requiring an emergency severity index of 2, indicating an Emergency Department triage upon presentation of less than 20 min (3). The data was derived from a systematic review of the medical literature.^a^

^a^A comprehensive search was conducted in PubMed (NLM), Embase (Ovid), Web of Science (Clarivate), CINAHL(EBSCOhost), and Criminal Justice (ProQuest) by a health sciences research librarian on December 15th, 2022. Using Covidence software,^14^ 417 titles and abstracts were independently reviewed by three reviewers, and 36 full-text papers for all potentially pertinent research were collected. The search used a combination of subject headings and keywords to find literature on the healthcare of youth in custody with chronic diseases in English; discussion and consensus-building were used to settle any disputes. Articles were included if any of the following conditions were met: (a) presented guidelines for the care of young people who are in custody and have conditions such as asthma, cystic fibrosis, insulin-dependent diabetes, epilepsy, cancer, or sickle cell disease, (b) examined the association between delayed treatment of these conditions while incarcerated and serious morbidity or mortality, met the inclusion criteria. Studies that failed to address one of the following conditions were excluded: (a) the healthcare of youth in custody or (b) did not report any chronic diseases. Using a descriptive thematic analysis methodology, data were extracted and compiled.

Youth in custody face numerous challenges in accessing healthcare services, such as limited resources, fragmented healthcare systems, and insufficient coordination between prison healthcare teams and external healthcare providers. Moreover, custodial facilities may lack the necessary infrastructure and specialized healthcare personnel to manage the time-sensitive acute medical needs of youth with chronic diseases. Despite the limitations of custodial facilities in healthcare delivery, the United States Supreme Court has held that “deliberate indifference” to the “serious medical needs” of incarcerated people violates the Constitution.^13^ We recognize the spirit of the court decision: individuals held in custody should receive the same level of care as if they were not in custody. However, the absence of clinical care guidelines that address the unique custodial setting has three immediate consequences. First, no actionable strategy is established to ensure youth with chronic disease exacerbation receive time-sensitive health care. Second, healthcare providers in these custodial facilities do not benefit from the evidence-based standards required for ongoing quality improvement strategies. Third, there is no accountability when the health care provider or system fails to deliver standard care in the custody setting. Unfortunately, the current absence of evidence-based guidelines can lead to a below-the-standard care approach for acutely ill youth experiencing a time-sensitive exacerbation of their chronic disease.

A unique challenge in implementing clinical standards for youth in custody is the absence of best practices for communicating between the healthcare staff in custody, the healthcare providers outside the community medical facility or practice (general pediatric providers and sub-specialty providers), and the youth and their parents. Given the wide range of healthcare needs of the incarcerated youth population, a clear and actionable communication strategy and a healthcare plan are required to ensure health equity for children with chronic disease; pediatric sub-specialists and pediatricians with expertise in carceral healthcare should be engaged in developing and implementing evidence-based guidelines and institution-specific protocols for care coordination. Strategies that include telemedicine, shared electronic health records, and multi-disciplinary care teams that involve specialty care providers inside facilities and in the community can facilitate seamless and timely communication between the two healthcare facilities. An important step towards this goal is ensuring pediatric sub-specialists understand the unique aspects of the custodial settings concerning their patient’s health and healthcare and acknowledge their role in championing the health of young people in custody. To our knowledge, few pediatric subspecialty training programs include learning objectives on managing acute exacerbations of the chronic care of children or adolescents held in custody.

We identified six chronic diseases requiring acute management while youth are held in custody. None of the professional society’s established developed age-specific evidence-based guidelines, Table 1. The list of chronic diseases that affect children and require timely management is far greater than the six chronic diseases that we identified, including the most common chronic condition in youth held in custody, mental illness. Given the prevalence and acute mental health care crisis in the United States, the subject matter is worthy of a stand-alone commentary. Furthermore, we did not include the importance of chronic illness treatment and ongoing modifiable healthcare plans for entry and exit from the carceral facility back into the community.

## Action agenda for sub-specialty professional societies

The care of children and adolescents with acute exacerbations of these six life-threatening chronic conditions (Type 1 diabetes, cystic fibrosis, sickle cell disease, epilepsy, cancer, and asthma) requires evidence-based guidelines and standards in custodial settings to prevent mortality and morbidity. We recommend all evidence-based guidelines include the management of children and adolescents held in custody and the inclusion of the stakeholders (former adolescents held in custody, their healthcare providers, and their parents) to ensure participation in developing these guidelines. Table 2 includes actionable priorities for medical sub-specialty professional societies. Implementation science indicates that if evidence-based guidelines are used, our children and adolescents with chronic diseases held in custody will have a demonstrable decrease in morbidity and mortality. We can and should do better to improve health equity in this invisible but prominent and vulnerable population of children and adolescents held in custody.

**Table 2:** Actionable Priorities for Medical Sub-Specialty Professional Societies.

Task	Example
Publish chronic disease-specific guidelines that address correctional settings	• American Diabetic Association • Asthma and Allergy Foundation of America • American Thoracic Society • Cystic Fibrosis Foundation • American Society of Hematology • American Epilepsy Society • American Cancer Society
Identify minimum medical equipment and contingent transfer strategies to address time-sensitive acute exacerbations of chronic diseases	• Management of hypoglycemia in youth with type 1 diabetes • Management of status asthmaticus in youth with asthma • Management of acute pulmonary exacerbation in youth with cystic fibrosis • Management of acute vaso-occlusive pain event, acute chest syndrome, stroke in youth with sickle cell disease • Management of a seizure • Management of acute illness, fever, or both in youth with cancer
Advocacy to ensure Medicaid continuity	• Advocacy to end “inmate exclusion” for health insurance coverage during prison
Monitoring and regulation of quality of care	• Advocacy to improve legislation on quality of care
Clinical care coordination from entry, duration of carceral care, and re-entry	• Advocacy to ensure health care continuity between correctional health provider/general pediatrician/subspecialty care and access to prescriptions on release.
Mandate care providers to offer parent engagement	• Telemedicine visits with parents as standard care

We have identified actionable medical care and advocacy priorities that subspecialty professional organizations can engage in to address the healthcare disparity in children and adolescents held in custody.

## Data Availability

Data sharing does not apply to this article as no datasets were generated or analyzed during the current study.
